# Supplementary material for: Neuronal Repressor REST Controls Ewing Sarcoma Growth and Metastasis by Affecting Vascular Pericyte Coverage and Vessel Perfusion
Source: Cancers (Basel). 2020 May 29;12(6):1405. doi: 10.3390/cancers12061405 (PMC7352345; doi:10.3390/cancers12061405)
Supplement: Supplementary file 1 [file cancers-12-01405-s001.pdf]

Supplemental Materials: Neuronal Repressor REST Controls Ewing Sarcoma Growth and Metastasis by Affecting Vascular Pericyte Coverage and Vessel Perfusion

Zhichao Zhou, Yuanzheng Yang, Fei Wang and Eugenie S. Kleinerman

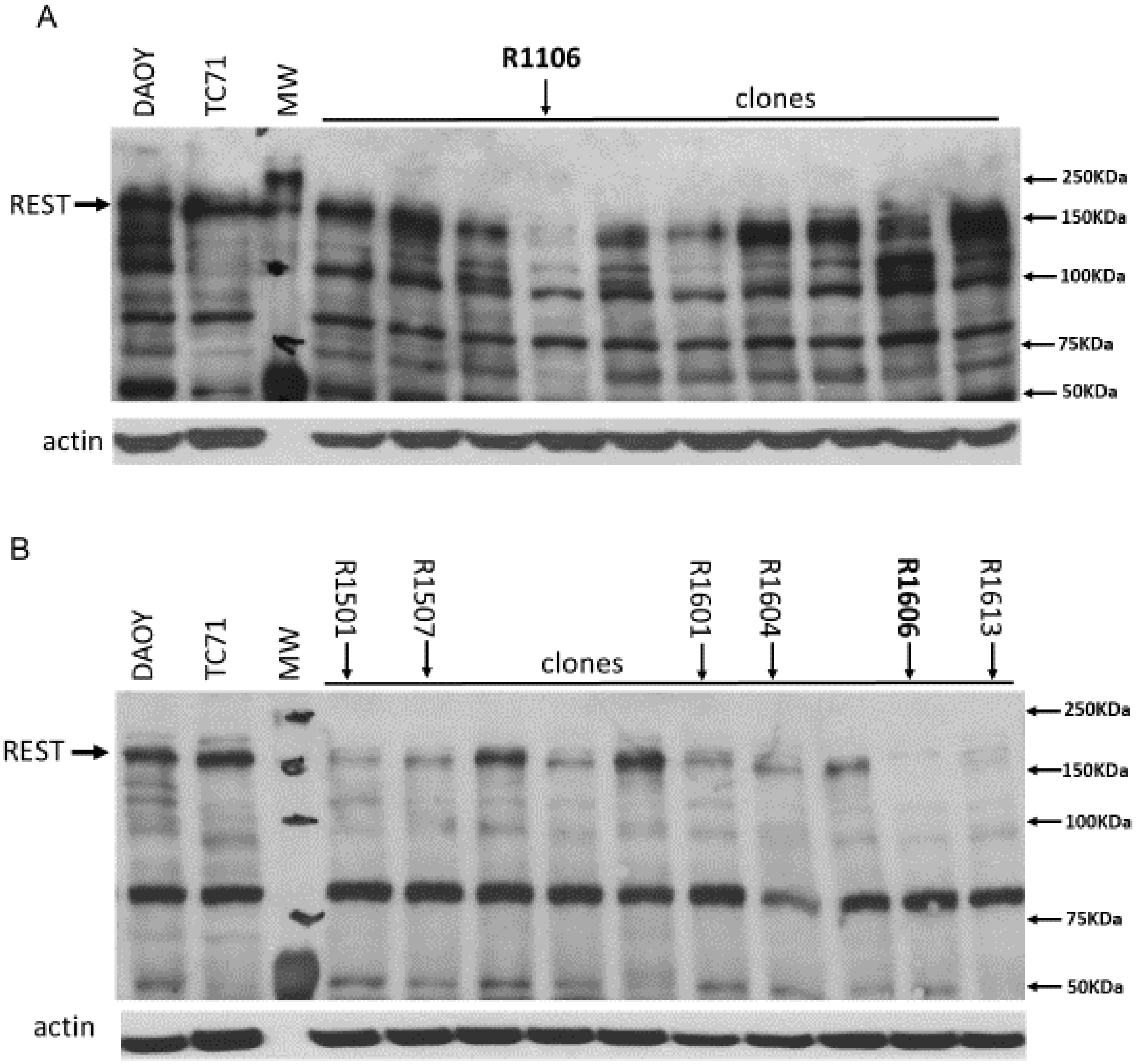

Figure 1. Representative WB analysis of CRISPER Clones from (**A**) single transfection of sgRNA and (**B**) double transfection of sgRNAs. Lanes pointed by arrow show REST knockout clones.

© 2020 by the authors. Licensee MDPI, Basel, Switzerland. This article is an open access article distributed under the terms and conditions of the Creative Commons Attribution (CC BY) license (http://creativecommons.org/licenses/by/4.0/).
